# Thoughts for Dentists

**Published:** 1860-01

**Authors:** Arthur C. Ford

**Affiliations:** Louisville, Jefferson Co., Ga.


### Correspondence.

### THOUGHTS FOR DENTISTS.

LOUISVILLE, JEFFERSON CO., GA., Dec. 15th, 1859.

MESSRS. EDITORS:—It was with such feelings of pleasure that I perused the article in your December number of the "Dental Register," by Dr. Wm. A. Pease, entitled "Thoughts for Dentists," that I could not refrain from exclaiming—

> "Two souls with but a single thought,
> Two hearts that beat as one."

(at least upon the subject contained therein.)

It has ever been to me a galling sore and heart-burn, to see so much envy, strife, rancour and hard feeling existing among my professional brethren, and it was this state of things that caused me, for three years after having commenced my course of study, to waver, as to whether I should permanently enlist in the Dental ranks or not, as I looked upon it as a disgrace to the profession, and to the members individually as gentlemen.

The great "apple of discord" is, as Dr. Pease justly remarks, the spirit of under-charging that exists among us, and permitting our patients to "beat us down" in our fees. This practice of "cheapening" ought never to be allowed, as it does and ever will degrade the dignity of any profession, to say nothing worse of it, and I, for one, have always, and ever will, "turn a deaf ear" to all propositions to "bunch" a jab, a so much-for-the-whole sort of a bargain, just as though they were brow-beating their tailor for a suit of clothes.

I would suggest, if you will permit me, through your columns, that the Dentists of every State have each and severally a Convention, to establish a Professional Fee Bill, and that they be widely published, so that the public may know, without inquiring of individual Dentists, the universal prices, and then every Dentist will have an equal opportunity to sink or swim, as the people will, most assuredly, when they have a settled price to pay, resort to the Dentist who has the best reputation for giving them value received.

Our country (the South,) has most need of some such movement, as there are so many itinerants passing through our midst, and, by the way, not unfrequently *preachers*, to which is occasionally added the *respectable callings* of horse-jockey, black-leg, etc., etc., who not only most grievously deceive the people in "*stuffing*" their teeth, but in by far the greater majority of cases, ruin their dupes' mouths beyond reparation, and thereby cause many to consider Dentistry a humbug. There ought to be a law in every State prohibiting all persons from practicing Dentistry who have neither graduated at a Dental college or are not able to produce a certificate of competency, and having studied at least two years with a Dentist or Dentists in good standing.

It is my fervent desire and greatest aim to do every thing in my humble power to exalt our profession in the eyes of the public, and I trust the profession *generally* will look at things of this nature in the same light as I do, and will put their "shoulders to the wheel," and roll off this stigma from our fair fame, and let us present an untarnished front in the ranks of the professions, let us be "second to none" in honor one to another, and to our patients severally.

Trusting that these few feeble lines may be suggestive of some step, that some abler and older head than mine may undertake, I remain,

Yours respectfully,

ARTHUR C. FORD.

